# Prevalence of Selected Chronic Conditions Among Children, Adolescents, and Young Adults in Acute Care Settings in Hawai‘i

**DOI:** 10.5888/pcd17.190448

**Published:** 2020-07-23

**Authors:** Tetine Sentell, So Yung Choi, Lance Ching, Michelle Quensell, L. Brooke Keliikoa, Émilie Corriveau, Catherine Pirkle

**Affiliations:** 1 Office of Public Health Studies, University of Hawai‘i at Mānoa, Honolulu, Hawai‘i; 2 JABSOM Biostatistics Core Facility, Department of Quantitative Health Sciences, University of Hawai‘i, Honolulu, Hawai‘i; 3 Surveillance, Evaluation and Epidemiology Office, Chronic Disease Prevention and Health Promotion Division, Hawai‘i State Department of Health, Honolulu, Hawai‘i

## Abstract

**Introduction:**

Chronic disease prevalence among young people is understudied generally and specifically for Native Hawaiian, Filipino, and Pacific Islander youth who are at high risk for these conditions. We determined the statewide prevalence of chronic diseases in acute care for those aged 5 to 29 years, including Native Hawaiians, Filipinos, and Pacific Islanders.

**Methods:**

We used Hawai‘i statewide inpatient and emergency department (ED) data across all payers from 2015–2016 to determine the presence of at least 1 of 5 chronic conditions (ie, asthma, hypertension, chronic kidney disease, diabetes, stroke) from 13,514 inpatient stays by 9,467 unique individuals and 228,548 ED visits by 127,854 individuals.

**Results:**

Twenty-eight percent of youth who were hospitalized and 12% with an ED visit had at least 1 chronic condition. Medicaid covered more than half of these visits. When comparing patients with and without a chronic condition, race/ethnicity, age group, and payer varied significantly in both inpatient and ED settings. Patients with a chronic condition were disproportionately Native Hawaiian, Filipino, and Pacific Islander; 32.3% of those with an inpatient chronic condition and 34.9% of those with an ED chronic condition were Native Hawaiian. Prevalence of chronic conditions among racial/ethnic groups varied significantly by age.

**Conclusion:**

Chronic diseases, including those more often seen in adulthood, are prevalent in young people in acute care settings in Hawai‘i, with notable disparities. These findings can help justify, guide, and support programs that are needed to address these changing epidemiological trends, which may be of particular interest for Medicaid.

**Summary**

**What is already known on this topic?**

Native Hawaiians, other Pacific Islanders, and Filipinos are rapidly growing populations in the United States, and these groups have a high prevalence of chronic disease risk factors. However, little is known about disease prevalence in these understudied populations.

**What is added by this report?**

We analyzed Hawai‘i inpatient and emergency department data to assess the statewide prevalence of chronic conditions in acute care for patients aged 5 to 29 years and found that patients who had a chronic condition were disproportionately Native Hawaiian, Filipino, and Pacific Islander.

**What are the implications for public health practice?**

Our findings can help justify, guide, and support programs to address disparities in chronic conditions among young people who are at high risk for these conditions.

## Introduction

Despite the epidemiological transition in the United States to chronic conditions at earlier ages, limited research has quantified population-based measures of chronic conditions for youth (1–3). Most US population-based surveillance instruments focus on people aged 18 or older (4). There is considerable interest in this topic around prevalence in the acute care specifically, because of the association of complex chronic conditions and increased costs and poorer outcomes in this setting (5,6). 

The World Health Organization has stated that young people today will live less healthy lives than their parents, in part because of higher rates and earlier onset of chronic diseases. Current evidence in the United States suggests that chronic disease among young people is a growing problem; however, prevalence of chronic diseases in this population is generally understudied (3). It is estimated that 1% to 3% of American children have hypertension, and stroke is among the top 10 causes of childhood death. One in 5 US children has obesity, and the prevalence of type 2 diabetes is projected to quadruple on a national level from 22,820 in 2010 to 84,131 in 2050 among people younger than 20 years (7). The consequences of a childhood diagnosis with certain chronic conditions are serious. A youth type 2 diabetes diagnosis entails a 23-fold increased risk of renal failure and a 39-fold increased risk of dialysis according to one large Canadian study of children aged 1 to 18 (8). Cardiometabolic risk profiles of US adolescents and young adults are also worrisome, with a growing prevalence of diabetes in adolescents, increasing diabetes-related complications in young adults, and approximately 1 in 5 adolescents and 1 in 4 young adults having prediabetes (3).

The risk profiles and prevalence of chronic disease in Native Hawaiians, Filipinos, and other Pacific Islander youth generally, and specifically in acute care settings, are understudied (9,10). These groups are among the fastest growing racial/ethnic populations in the United States and experience substantial chronic disease disparities at earlier ages, many of which may be established during childhood (11). Research in adults indicates that these populations experience asthma, diabetes, hypertension, stroke, and heart failure at higher rates and earlier ages compared with many other racial/ethnic groups (9–12). Research in youth shows disproportionate risk factors for chronic conditions in these populations, including higher rates of obesity, less physical activity, poorer nutrition, and less access to both health care and healthy food options (13–15). These risk factors for chronic disease are associated with poorer health outcomes later in life as well as poor life course trajectories (16,17).

Programs to address chronic disease in youth are urgently needed, but data are needed to understand the scope of the issue, to justify funding prioritization, and to tailor programs to meet demonstrated need, especially for Native Hawaiian, Filipino, and other Pacific Islander communities (3,13,17–19). Prevalence in the acute care setting can identify many of the most severe and costly outcomes. The goal of this study was to determine the prevalence in Hawai‘i of chronic diseases in acute care (inpatient and emergency department [ED]) for those aged 5 to 29 years, including Native Hawaiian, Filipino, and Pacific Islander populations. We used a broad definition of youth, because understanding patterns across the early life course through the distinct periods of vulnerability of adolescence and young adulthood are critical for designing effective, holistic public health approaches (20). Using this definition was important because of our focus on Native Hawaiian, Filipino, and Pacific Islander populations, as the health vulnerabilities of early adulthood are impactful for racial/ethnic minorities (20). 

## Methods

### Population and sample

We used Hawai‘i statewide 2015–2016 data from inpatient (N = 31,400) and ED (N = 261,890) visits across all payers for those aged 5–29 years. Data were from the Hawai’i Health Information Corporation (HHIC), the source for Hawaii’s Healthcare Cost and Utilization Project data during the study period.

We excluded visits with unknown race/ethnicity (inpatient, 514; ED, 4,700), then unknown county of residence/non-Hawai‘i residents (inpatient, 740; ED, 15,497), then excluded those with a pregnancy code (inpatient, 16,632; ED, 13,145). Pregnancy is the most common reason for hospitalization in the United States (21), but we did not want pregnancy-related visits in early adulthood and the associated sex differential to influence findings. After exclusions, the study samples were 13,514 inpatient hospitalizations by 9,467 unique individuals and 228,548 emergency department visits by 127,854 unique individuals.

### Measures

Data elements included a unique patient identifier, patient race/ethnicity, age, sex, insurer, and primary and secondary *International Classification of Diseases, Clinical Modification* (ICD-CM) diagnostic codes. We considered the presence of at least 1 of 5 targeted chronic conditions (asthma, hypertension, chronic kidney disease [CKD], diabetes, stroke). We focused on these chronic diseases because of their associated high cost and/or state prevalence across the life span and notable disparities (10–13). To identify these conditions, we used ICD-9-CM codes (relevant through September 2015) and ICD-10-CM codes (relevant after September 2015) available for 1 primary and up to 37 secondary categories. The codes used to identify each disease category are available at https://manoa.hawaii.edu/publichealth/research-teams/hhiet/evaluation-projects. Given the change from ICD-9-CM to ICD-10-CM, we also considered changes in prevalence of our target chronic conditions across this transition. A supplementary table with this information is also available at the website above. Consistent with literature on the transition in adulthood (22), we found that trends remained stable and thus report combined data here.

Race/ethnicity was self-reported primary race/ethnicity at intake and was consistently collected across all hospitals in Hawaiʻi following standardized protocols and quality assurance (23). For those who provided multiple races/ethnicities, we followed a standard protocol (24). If Native Hawaiian was one of the reported races/ethnicities, we coded it as “part Hawaiian.” “Native Hawaiian” includes patients who reported full and part Hawaiian. If a nonwhite race/ethnicity was given with white race/ethnicity, the nonwhite race/ethnicity was coded. If more than one nonwhite race/ethnicity was given, the first one was coded.

To understand the general costs of these visits including chronic conditions to the state, we applied a standard cost-to-charge ratio to the inpatient charge data for cost estimates (25). These metrics convert hospital charges to estimates of actual hospital costs using data from hospital accounting reports from the Centers for Medicare & Medicaid Services. Because there is no standard, publicly available cost-to-charge ratio for ED data, we report charges only for ED.

### Statistical analysis

For patients with multiple visits during the 2-year study period, we used patient characteristics at first visit, with one exception. If patients had a visit that included a chronic disease code at any point during the study period, they were considered in the “chronic disease” category. These determinations were made separately in each sample (ie, inpatient and ED); we did not track individual chronic disease status across inpatient and ED visits. Specifically, 819 inpatient hospitalizations and 18,981 ED visits by individuals without a chronic disease code assigned at that time had a subsequent visit with a chronic disease code.

We used descriptive statistics for each patient to examine each of the 5 target chronic conditions (categories not mutually exclusive). We used χ^2^ tests and 2-sample *t* tests to compare patients with at least 1 chronic disease to those without a chronic disease during the study period (categories mutually exclusive). We also considered variation in percentages of any of the focal chronic diseases (yes or no) overall by race/ethnicity across age groups.

All analyses were done in R version 3.6.0 (R Core Team). This study was deemed exempt by the University of Hawaiʻi institutional review board.

## Results

### Inpatient stays

Of the 9,467 unique individuals who were hospitalized, 28.1% (2,659) had at least 1 of the 5 chronic diseases; 16.7% had asthma, 7.7% had hypertension, 1.7% had CKD, 8.3% had diabetes, and 1.1% had a stroke (Table 1). Native Hawaiians made up a high percentage of those hospitalized with asthma (38.2%). Other Pacific Islanders (27.4%) and Filipinos (22.9%) made up a high percentage of those with CKD, and of those hospitalized with a coded diabetes diagnosis (785), 69.7% had type 2 or other diabetes (vs type 1 diabetes).

**Table 1:** Inpatient Data for Children/Young Adults Aged 5–29 Years, Hawai‘i Health Information Corporation (N = 9,467 Unique Individuals), January 2015–December 2016 a^,^^b^

Characteristic	Asthma	Hypertension	CKD	Any Diabetes	Stroke	Total With Any Chronic Disease	Total With No Chronic Disease	*P* Value^c^	All Inpatient
**Total unique individuals**	1,577 (16.7)	726 (7.7)	157 (1.7)	785 (8.3)	100 (1.1)	2,659 (28.1)	6,808 (71.9)	NA	9,467
**Mean no. of visits by unique individuals (SD) [range]**	1.3 (1.2) [1–26]	1.6 (1.8) [1–20]	2.4 (2.9) [1–24]	1.7 (2.3) [1–25]	1.1 (0.5) [1–4]	1.5 (1.6) [1–26]	1.3 (1.1) [1–23]	NA	1.4 (1.5) [1–30]
**Study Sample Characteristics at First Visit**									
**Race/ethnicity**									
Chinese	34 (2.2)	18 (2.5)	—a	16 (2.0)	—a	60 (2.3)	185 (2.7)	<.001	243 (2.6)
Filipino	238 (15.1)	129 (17.8)	36 (22.9)	123 (15.7)	15 (15.0)	431 (16.2)	981 (14.4)		1,410 (14.9)
Native Hawaiian	602 (38.2)	203 (28.0)	33 (21.0)	240 (30.6)	24 (24.0)	858 (32.3)	1,505 (22.1)		2,363 (25.0)
Japanese	102 (6.5)	52 (7.2)	15 (9.6)	46 (5.9)	—a	176 (6.6)	540 (7.9)		716 (7.6)
Other Pacific Islander	170 (10.8)	153 (21.1)	43 (27.4)	152 (19.4)	16 (16.0)	392 (14.7)	805 (11.8)		1,198 (12.7)
Other race/ethnicity	136 (8.6)	65 (9.0)	—a	67 (8.5)	13 (13.0)	239 (9.0)	799 (11.7)		1,042 (11.0)
White	295 (18.7)	106 (14.6)	16 (10.2)	141 (18.0)	20 (20.0)	503 (18.9)	1,993 (29.3)		2,495 (26.4)
**Age, y**									
5–9	308 (19.5)	26 (3.6)	—a	48 (6.1)	—a	375 (14.1)	714 (10.5)	<.001	1,092 (11.5)
10–14	227 (14.4)	42 (5.8)	—a	82 (10.5)	—a	316 (11.9)	847 (12.4)		1,163 (12.3)
15–19	292 (18.5)	75 (10.3)	—a	125 (15.9)	13 (13.0)	439 (16.5)	1,522 (22.4)		1,962 (20.7)
20–24	345 (21.9)	196 (27.0)	50 (31.7)	199 (25.4)	20 (20.0)	619 (23.3)	1,797 (26.4)		2,416 (25.5)
25–29	405 (25.7)	387 (53.3)	86 (54.8)	331 (42.2)	50 (50.0)	910 (34.2)	1,928 (28.3)		2,834 (29.9)
**Male sex**	824 (52.3)	436 (60.1)	80 (51.0)	390 (49.7)	58 (58.0)	1,427 (53.7)	3,683 (54.1)	.72	5,110 (54.0)
**Payer**									
Medicaid/Quest	934 (59.2)	393 (54.1)	66 (42.0)	419 (53.4)	39 (39.0)	1,478 (55.6)	3,091 (45.4)	<.001	4,565 (48.2)
Private Insurance	523 (33.2)	246 (33.9)	52 (33.1)	292 (37.2)	49 (49.0)	942 (35.4)	2,780 (40.9)		3,723 (39.3)
Other	120 (7.6)	87 (12.0)	39 (24.8)	74 (9.4)	12 (12.0)	239 (9.0)	937 (13.8)		1,179 (12.5)
**County (residence)**									
Oahu	1,022 (64.8)	504 (69.4)	121 (77.1)	552 (70.3)	61 (61.0)	1,776 (66.8)	4,602 (67.6)	.47	6,379 (67.4)
Other	555 (35.2)	222 (30.6)	36 (22.9)	233 (29.7)	39 (39.0)	883 (33.2)	2,206 (32.4)		3,088 (32.6)
**County (hospital)**									
Oahu	1,128 (71.5)	554 (76.3)	131 (83.4)	609 (77.6)	75 (75.0)	1,966 (73.9)	5,130 (75.4)	.16	7,090 (74.9)
Other	449 (28.5)	172 (23.7)	26 (16.6)	176 (22.4)	25 (25.0)	693 (26.1)	1,678 (24.7)		2,377 (25.1)
**Comorbid diabetes**	108 (6.9)	153 (21.07)	37 (23.6)	556 (70.8)	—a	520 (19.6)	0 (0.00)	<.001	482 (5.1)
**Mean age (SD) at first visit**	18.0 (7.5)	23.3 (5.7)	23.7 (5.1)	21.5 (6.4)	22.0 (7.1)	19.7 (7.4)	19.5 (6.7)	.48	19.6 (6.9)
**Total cost adjusted with CTC ratio, $**	66,614,415	57,819,857	20,529,954	52,161,763	10,823,761	150,029,971	274,550,553	NA	450,640,122

Abbreviations: CKD, chronic kidney disease; CTC, Cost to Charge Ratio; NA, not applicable; SD, standard deviation.

a Per data use agreement rules of the Hawai‘i Health Information Corporation, we cannot release or disclose information in which the number of observations (ie, individual discharge records) in any given cell of tabulated data are less than or equal to 10. Values are number (%) unless otherwise indicated.

b The chronic disease columns (asthma, hypertension, CKD, any diabetes, stroke) are not mutually exclusive. Columns with the *P* value comparisons “any chronic disease” vs “no chronic disease” during the study period are mutually exclusive.

c For comparison of any chronic disease and no chronic disease.

Of those hospitalized with any focal chronic disease during the study period, 32.3% were Native Hawaiian, 18.9% white, 16.2% Filipino, and 14.7% Other Pacific Islander; 14.1% of patients with any chronic disease were aged 5 to 9 years, and 34.2% were aged 25 to 29 years. Those with a chronic disease varied significantly (*P* < .001) from those without a chronic disease by race/ethnicity, age, and payer. Of patients hospitalized with a chronic disease, a larger percentage were Native Hawaiian, Filipino, and Pacific Islander; in the youngest and oldest age categories; and covered by Medicaid.

**Figure 1:**
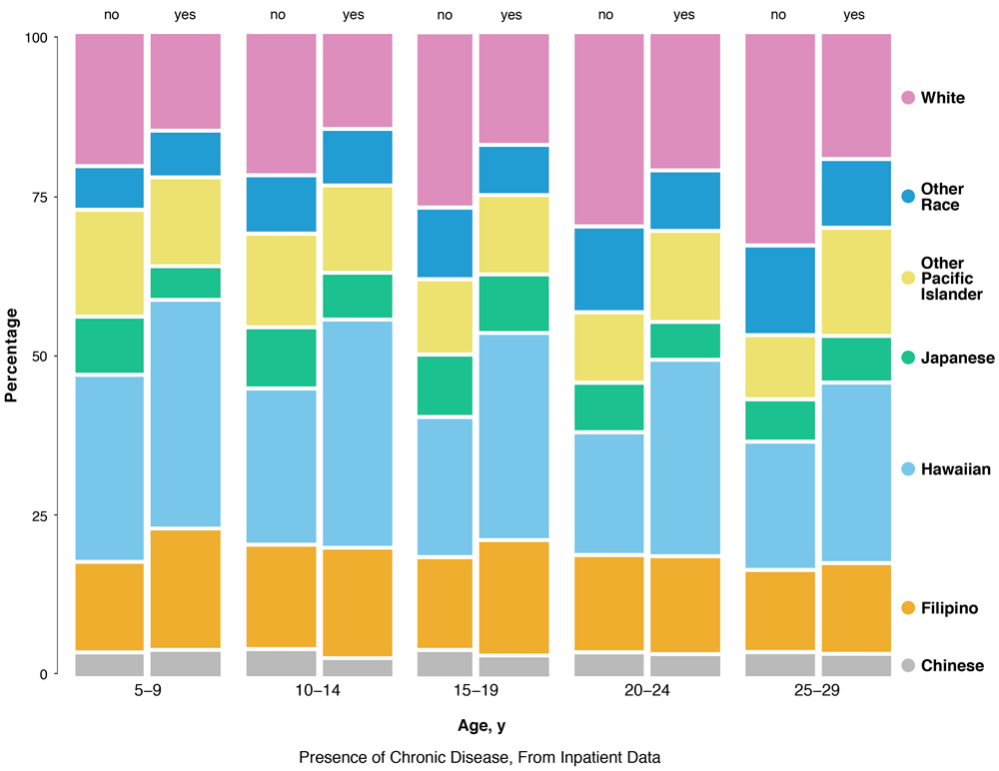
Three-way mosaic plot of chronic disease status of patients, by race/ethnicity and age, inpatient data for children and young adults aged 5–29 years from the Hawai‘i Health Information Corporation, January 2015 through December 2016 (N = 9,467 unique individuals).

The proportion of patients with any chronic disease varied significantly (*P* value range from <.001 to .01) by race/ethnicity across all age groups (Figure 1). The proportion of patients hospitalized with a focal chronic condition who were Native Hawaiian and Filipino decreased as age increased. For example, Native Hawaiians comprised 37% of patients with chronic disease in the youngest age groups (5–9 years and 10–14 years) but only 28% in the oldest (25–29 years). By contrast, the proportion of other Pacific Islanders among those hospitalized with a focal chronic condition increased from 13.9% among those aged 5–9 years to 17.0% of those aged 25–29 years. The proportion of white and Japanese patients among those hospitalized with a chronic condition also increased as age increased.

**Table:** 

Race/Ethnicity	Age, y									
	5–9		10–14		15–19		20–24		25–29	
	No	Yes	No	Yes	No	Yes	No	Yes	No	Yes
White	21.3	15.5	22.8	15.2	28.1	17.8	31.2	22.0	34.3	20.1
Other race	6.4	6.9	8.9	8.5	11.0	7.5	13.4	9.2	14.0	10.5
Other Pacific Islander	16.8	13.9	14.6	13.6	11.6	12.3	10.9	14.2	9.8	17.0
Japanese	8.8	4.8	9.3	7.0	9.5	8.9	7.4	5.5	6.2	6.9
Hawaiian	30.0	36.8	24.9	36.7	22.3	33.3	19.4	31.5	20.3	28.9
Filipino	14.1	19.2	16.4	17.4	14.5	18.2	15.2	15.3	12.8	14.2
Chinese	2.5	2.9	3.1	1.6	3.0	2.1	2.6	2.3	2.6	2.3

The total cost for the hospitalizations with at least 1 chronic disease was more than $450 million in 2 years. Medicaid was the primary payer for 55.6% of these hospitalizations.

### Emergency department visits

Of the 127,854 individuals with an ED visit, 12.8% were for a patient with at least 1 of the 5 focal chronic diseases; 10.7% had asthma, 1.5% had hypertension, less than 1% had CKD, 1.3% had diabetes, and less than 1% had a stroke (Table 2). Of those with a coded diabetes diagnosis (1,634), 87.2% had type 2 or other diabetes (vs type 1 diabetes). Of those with an ED visit who had at least 1 chronic disease, 34.9% identified as Native Hawaiian, 17.0% white, 18.0% Filipino, and 10.1% other Pacific Islander. Medicaid was the primary payer for 53.5% of these ED visits. Twenty-one percent were for those aged 5 to 9 years, and 24.7% were for those aged 25 to 29 years. Those with a chronic disease varied significantly (*P* < .001) from those without a chronic disease by race/ethnicity, age group, and payer; those with a chronic disease had a higher percentage of Native Hawaiians and people covered by Medicaid. The total charge for ED visits with at least one chronic condition coded was more than $60 million in the 2-year period.

**Table 2:** Emergency Department Data for Children/Young Adults Aged 5–29 Years From the Hawai‘i Health Information Corporation (N = 127,854 Unique Individuals), January 2015–December 2016 a^,^^b^

Characteristic	Asthma	Hypertension	CKD	Any Diabetes	Stroke	At Least 1 Chronic Disease	No Chronic Disease	*P* Value^c^	All ED
**Total unique individuals**	13,720 (10.7%)	1,960 (1.5%)	119 (0.1%)	1,634 (1.3%)	57 (0.04%)	16,412 (12.8%)	111,442 (87.2%)	NA	127,854
**Mean no. of per-person visits (SD) [range]**	1.6 (1.7) [1–50]	2.0 (2.9) [1–53]	3.3 (5.4) [1–38]	2.2 (3.3) [1–64]	1.1 (0.5) [1–4]	1.7 (2.1) [1–65]	1.6 (1.4) [1–62]	NA	1.8 (2.0) [1–171]
**Study Sample Characteristics at First Visit**									
**Race/ethnicity**									
Chinese	247 (1.8)	39 (2.0)	—a	28 (1.7)	0 (0.00)	300 (1.8)	2,783 (2.5)	<.001	3,076 (2.4)
Filipino	2,425 (17.7)	378 (19.3)	29 (24.4)	294 (18.0)	—a	2,952 (18.0)	19,083 (17.1)		22,017 (17.2)
Native Hawaiian	5,057 (36.9)	585 (29.8)	29 (24.4)	473 (29.0)	18 (31.6)	5,729 (34.9)	25,828 (23.2)		31,585 (24.7)
Japanese	840 (6.1)	134 (6.8)	—a	107 (6.6)	—a	1,038 (6.3)	8,027 (7.2)		9,066 (7.1)
Other Pacific Islander	1281 (9.3)	251 (12.8)	24 (20.2)	261 (16.0)	—a	1,665 (10.2)	13,189 (11.8)		14,846 (11.6)
Other race/ethnicity	1,655 (12.1)	236 (12.0)	14 (11.8)	157 (9.6)	—a	1,944 (11.8)	14,078 (12.6)		16,019 (12.5)
White	2,215 (16.1)	337 (17.2)	14 (11.8)	314 (19.2)	15 (26.3)	2,784 (17.0)	28,454 (25.5)		31,245 (24.4)
**Age, y**									
5–9	3,319 (24.2)	20 (1.0)	—a	76 (4.7)	—a	3,399 (20.7)	22,515 (20.2)	<.001	26,002 (20.3)
10–14	2,286 (16.7)	27 (1.4)	—a	139 (8.5)	—a	2,420 (14.8)	17,803 (16.0)		20,224 (15.8)
15–19	2,578 (18.8)	141 (7.2)	—a	259 (15.9)	—a	2,891 (17.6)	20,722 (19.0)		23,643 (18.5)
20–24	2,902 (21.2)	560 (28.6)	32 (26.9)	432 (26.5)	17 (29.8)	3,643 (22.2)	25,508 (22.9)		29,193 (22.8)
25–29	2,635 (19.2)	1,212 (61.8)	68 (57.1)	728 (44.6)	20 (35.1)	4,059 (24.7)	24,894 (22.3)		28,792 (22.5)
**Male sex**	7,148 (52.1)	1,068 (54.5)	54 (45.4)	657 (40.2)	30 (52.6)	8,475 (51.6)	56,563 (50.8)	.03	65,039 (50.9)
**Payer**									
Medicaid/Quest	7,617 (55.5)	913 (46.6)	42 (35.3)	794 (48.6)	16 (28.1)	8,777 (53.5)	45,091 (40.5)	<.001	53,782 (42.1)
Private insurance	4,816 (35.1)	704 (35.9)	35 (29.4)	615 (37.6)	34 (59.7)	5,895 (35.9)	46,588 (41.8)		52,436 (41.0)
Other	1,287 (9.4)	343 (17.5)	42 (35.3)	225 (13.8)	—a	1,740 (10.6)	19,763 (17.7)		21,636 (16.9)
**County (residence)**									
Oahu	8,490 (61.9)	1,337 (68.2)	85 (71.4)	1,135 (69.5)	38 (66.7)	10,409 (63.4)	75,760 (68.0)	<.001	86,174 (67.4)
Other	5,230 (38.1)	623 (31.8)	34 (28.6)	499 (30.5)	19 (33.3)	6,003 (36.6)	35,682 (32.0)		41,680 (32.6)
**County (hospital)**									
Oahu	8,526 (62.1)	1,342 (68.5)	88 (74.0)	1,143 (70.0)	38 (66.7)	10,454 (63.7)	75,821 (68.0)	<.001	86,284 (67.5)
Other	5,194 (37.9)	618 (31.5)	31 (26.1)	491 (30.1)	19 (33.3)	5,958 (36.3)	35,621 (32.0)		41,570 (32.5)
**Has comorbid diabetes**	216 (1.6)	272 (13.9)	25 (21.0)	1,438 (88.0)	—a	1,353 (8.2)	0	<.001	1,033 (0.8)
**Mean (SD) age at first visit, y**	16.6 (7.5)	24.8 (4.0)	23.7 (5.5)	22.1 (6.0)	20.6 (6.9)	17.8 (7.6)	17.5 (7.4)	<.001	17.5 (7.4)

Abbreviations: CKD, chronic kidney disease; ED, emergency department; NA, not applicable; SD, standard deviation.

a Per data use agreement rules of the Hawai‘i Health Information Corporation, we cannot release or disclose information where the number of observations (ie, individual discharge records), in any given cell of tabulated data are less than or equal to 10. Values are number (%) unless otherwise indicated.

b Columns with the *P* value comparisons “any chronic disease” vs “no chronic disease” during the study period are mutually exclusive.

c For comparison of total any chronic disease to no chronic disease.

**Figure 2:**
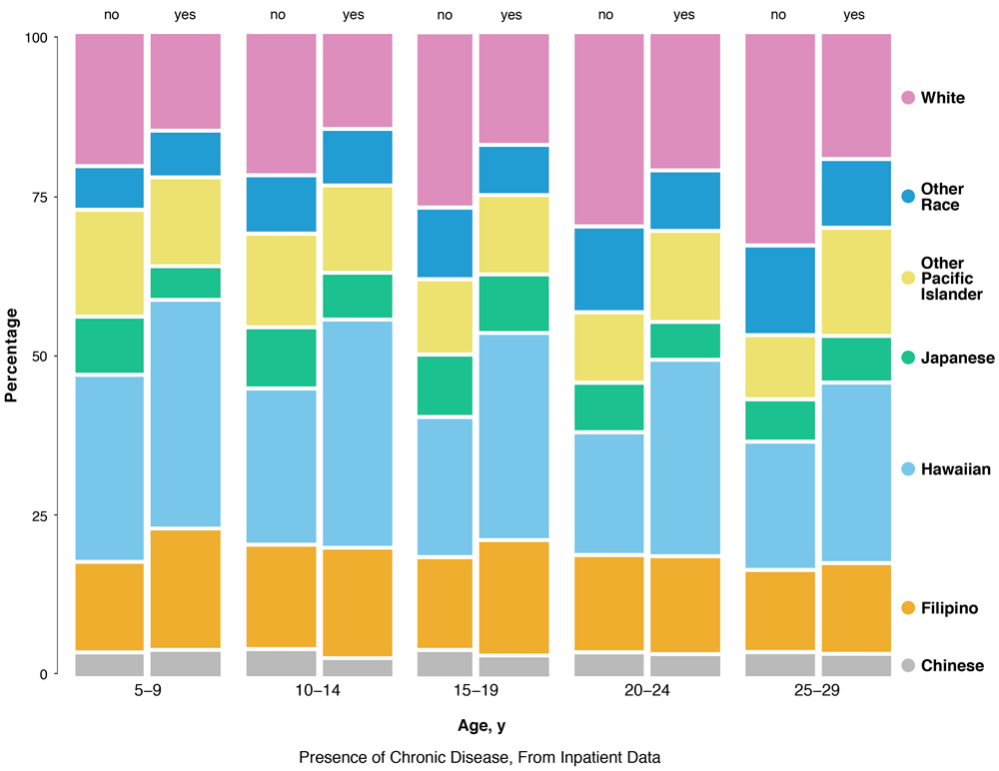
Three-way mosaic plot of chronic disease status of patients, by race/ethnicity and age, from emergency department data for children/young adults aged 5–29 years from the Hawai‘i Health Information Corporation, January 2015 through December 2016 (N = 127,854 unique individuals).

As in the inpatient setting, the proportion of ED patients with any chronic disease varied significantly by race/ethnicity across all age groups (Figure 2). The proportion of ED patients with a focal chronic condition who were Native Hawaiian and Filipino decreased as age increased. Distinct from the inpatient setting, the proportion of other Pacific Islanders among ED patients with a chronic condition also decreased over time. The proportion of ED patients with a chronic disease who were white and Japanese increased as age increased.

**Table:** 

Race/Ethnicity	Age, y									
	5–9		10–14		15–19		20–24		25–29	
	No	Yes	No	Yes	No	Yes	No	Yes	No	Yes
White	21.2	13.6	20.7	14.9	22.5	16.1	29.2	18.6	31.6	20.1
Other race	11.7	9.8	11.5	11.2	12.3	12.2	13.9	13.4	13.2	12.2
Other Pacific Islander	13.5	11.1	12.1	10.3	11.8	9.2	11.2	9.2	10.7	10.7
Japanese	7.1	4.9	8.4	6.0	8.0	8.1	6.4	6.6	6.6	6.2
Hawaiian	25.9	38.3	26.5	37.3	24.6	35.2	20.5	33.5	19.9	31.7
Filipino	17.9	20.4	18.4	19.0	18.0	17.3	16.5	16.8	15.5	16.9
Chinese	2.7	1.9	2.4	1.2	2.8	1.8	2.2	1.8	2.4	2.1

## Discussion

Chronic diseases, including those more often seen in adulthood, were prevalent in children, adolescents, and young adults in the acute care setting in Hawai‘i. These rates were higher overall than rates in other locations (1–4). Native Hawaiians in both inpatient and ED settings had a higher prevalence of chronic conditions than other racial/ethnic groups. Filipinos and other Pacific Islanders were also hospitalized with chronic conditions at higher percentages than other racial/ethnic groups.

An important pattern that emerged was the prevalence of these chronic conditions across multiple ages from early childhood to young adulthood. With the exception of patients aged 5 to 9 years, we found an almost dose–response-type relationship between increasing age and prevalence of chronic conditions in acute care. This finding indicates the need for targeted prevention efforts in early childhood as well as chronic disease management programs designed for youth, adolescents, and young adults, all of whom have distinct needs and vulnerabilities (16–20). Community clinical linkages to acute care settings may be a useful response, and these results can help to justify and target such efforts.

Other patterns in the acute care setting emerged across age groups by race/ethnicity. Native Hawaiians made up the largest proportion of hospitalized and ED patients with a focal chronic condition across all age groups, but the proportions decreased over time. This may represent early risk for Native Hawaiians and indicate relevant times for intervention. Increasing proportions at later ages were also noteworthy, especially for hospitalized Pacific Islanders. Some of these differences may have resulted from disease trends by race/ethnicity (eg, asthma vs CKD risk) and/or patterns of migration in and out of Hawai‘i by various population groups at different ages. Future work should examine this in more detail. Native Hawaiians appear to be at particular risk across all ages, as their proportion with chronic conditions in acute care was notably higher than the proportion in the state’s population. According to 2016 American Community Survey estimates, those identifying as Native Hawaiian alone or any racial/ethnic combination comprise 22.9% of Hawaii’s population aged 5 to 17 years; 10.4% aged 18 to 25 years; and 15.7% of those 25 aged 34 years (26).

Prevention and management of these chronic diseases and addressing these disparities is critical in general but particularly for Medicaid, which covers one third of children in Hawai‘i (27) and which paid for a disproportionate share of these acute care visits. Medicaid was the payer for 48% of the impatient stays and for 56% of those with at least 1 chronic condition, while private insurance paid for a smaller proportion of those hospitalized with at least 1 chronic condition (35%) compared with all hospitalizations (39%). Those who were hospitalized possibly were placed on Medicaid during the hospitalization, but Medicaid should have an interest in programs to reduce or reverse these trends in chronic disease given these results. Also noteworthy was that more than half of the hospitalized diabetes in this group of young people was for type 2 or other diabetes, not type 1. Type 2 diabetes is underdiagnosed generally and in youth, so actual risk may have been higher (31,32). Research on youth aged 18 or younger in Canada documented a high burden of renal complications and poor renal and overall survival associated with youth-onset type 2 diabetes (8).

Type 2 diabetes, CKD, hypertension, asthma, and many strokes are preventable conditions with existing evidence-based programs to prevent or reduce risk (18,19). Because many chronic conditions are largely irreversible, childhood onset can require careful lifelong management. Otherwise, the patient is likely to experience significant morbidity, often during their most economically productive years of life (3,7), which can have individual and family-related consequences. For example, children with chronic diseases may have high rates of school absenteeism that negatively affects their scholastic achievement, while their caregivers may miss work to care for them, which can negatively affect household income and ability to pay for medical care and disease management services (3,18,19). As children grow into adolescence and young adulthood, these risks must be considered in relation to transitions from parental supervision to autonomy in nutritional intake, health care access, health-risk behaviors, and other factors (20). These present unique stresses and vulnerabilities as well as new opportunities to intervene, including across new media platforms and social networks (20).

Study results can help justify and develop appropriate evidence-based interventions to address these emergent health issues. Culturally grounded interventions, such as those that include community and family, incorporate traditional dance, connect to the land or ocean, and consider spirituality may be relevant for Native Hawaiian, other Pacific Islander, and Filipino populations (28). Many effective programs, such as the Diabetes Prevention Program, are created for adults (29). The prediabetes screener promoted by the Centers for Disease Control and Prevention is geared toward older adults (30). Better understanding of costs and trends in children, adolescents, and young adults is important to justify efforts to modify such programs to meet the unique needs of youth and young adults. Acute care is a particularly expensive setting, and understanding such trends can provide justification for action. Understanding the full burden of chronic disease in younger populations can change policy and reimbursement for chronic disease management programs, which will become urgent if present trends continue. Acute health care use for these conditions has immediate cost-related consequences as well as long-term implications (1–3,10–13). These data are also important for estimating current and future burden of disease and associated costs.

This study has strengths and limitations. One strength is that the HHIC data are a census of all hospitalizations for the state and not a sample, which makes analyses of even small numbers more precise. However, these data are health care utilization data, which cannot be used to determine true prevalence of a condition in the general population; this was why we focused on providing prevalence estimates in the acute care setting specifically. It is likely that results from this study only capture the “tip of the iceberg,” as underdiagnosis of chronic conditions in the American health system is well-documented; therefore, it is reasonable to expect higher levels of underdiagnosis for traditionally low-risk youth (1–4). Patients who receive acute health care may vary from the general state population, so future population-based research would be useful to fully understand risk. Etiological associations for study outcomes and other related topics, including obesity, mental health, and cancer would also be useful.

Another limitation is that the ICD coding transition may have affected our study findings. In addition, the hospitalizations and ED visits among youth with a code for at least 1 of the 5 chronic diseases were not necessarily primarily due to 1 of these 5 focal diseases, and in many cases were comorbidities. Nevertheless, the numbers were high. We also did not have cost-to-charge data for the ED and thus overestimated the actual direct costs. The indirect costs of both the inpatient and ED stays for these children is larger, given the childcare, educational, and early workforce implications.

Hawai‘i is a unique location that well represents the multicultural demographics that continue to develop in much of the United States, including California and New York. Hawai‘i also provided a large enough sample size to investigate differences in heterogeneous Asian and Pacific Islander groups, which are generally understudied in the United States (9). The data collection protocol prioritizes classifying Native Hawaiians over non-Hawaiian and nonwhite populations over white populations, which may increase evidence of disparities. However, if this research serves to build meaningful public health programs to target demonstrated need across diverse communities this will only promote stronger and more equitable health outcomes. Patient chronic disease status changes over time, and we assumed this would only go one direction (diagnosis and progression); for this reason, we coded anyone who developed one of our focal chronic conditions during the 2 years as having the disease. Even if a disease was not noticed early on, it may have been undiagnosed. Stroke is not a chronic condition, but because it is a significant complication it was included. Although we considered chronic conditions overall by race/ethnicity and age, distinct diagnoses have unique patterns. For instance, the proportion of patients with hypertension varied substantially by age group (from 3.6% in those aged 5–9 to 53.3% in those age 25–29); similar patterns were observed for CKD and stroke, while the pattern for asthma differed. Interactions of conditions by age, race/ethnicity, and sex may show differential risk at certain critical periods. Considering household income, location of residence, and many other factors not available in this administrative data would be useful.

In conclusion, we found that chronic diseases, including those more often seen in adulthood, are prevalent in young people in acute care settings in Hawai‘i, with trends indicating disparities for Native Hawaiians, Filipinos, and Other Pacific Islanders. Programs are needed to address and reverse these changing epidemiological trends.
